# Open Versus Laparoscopic Ventral Hernia Repair: A Randomized Clinical Trial

**DOI:** 10.7759/cureus.20490

**Published:** 2021-12-17

**Authors:** Chirag Pereira, Rakesh Rai

**Affiliations:** 1 General Surgery, Father Muller Medical College and Hospital, Mangalore, IND

**Keywords:** post operative pain, ventral hernia, open ventral hernia repair, laparoscopic ventral hernia repair, hernia

## Abstract

Introduction

With the advancement in technology as well as surgical techniques, laparoscopic ventral hernia repair (LVHR) is more commonly being performed as compared to open repair in various centres throughout the world. Our study aimed to compare the short-term operative outcomes between LVHR and open repair.

Materials and methods

Sixty patients diagnosed with noncomplicated ventral hernias were included in this prospective study and were randomly divided into the laparoscopic group and the open group. The two groups were compared to evaluate operative time, postoperative pain, length of hospital stay and time taken to return to normal activity. A p-value of less than 0.05 was considered to be statistically significant.

Results

Mean operative time was longer in LVHR (116 min) as compared to open repair (67 min)(p<0.01). Patients experienced more pain on the first and seventh postoperative days in the open group (p<0.01) and they also had a longer duration of hospital stay as compared to the laparoscopic group (6.23 ± 0.35 vs 2.17 ± 1.12 days, p = 0.02). Patients in the laparoscopic group returned to normal activity faster as compared to the open group (1.47 ± 0.11 vs 2.87 ± 0.34, p<0.01).

Conclusion

LVHR carries a significant advantage over open hernia repair, especially in terms of reduced postoperative pain, duration of hospital stay, and early resumption of normal activity.

## Introduction

Ventral hernia is a protrusion of abdominal contents through a defect in the abdominal wall, with the exception of femoral and inguinal hernias [1]. Ventral hernias can be classified as primary, secondary, or based on the site they develop on the abdominal wall. Primary hernias include umbilical, epigastric, and hypogastric hernias while secondary ventral hernias occur following surgery, hence also referred to as incisional hernia [2].

The two main approaches in managing ventral hernias are open and laparoscopic surgery. William and Leblack first reported treatment of ventral hernia by laparoscopy in 1993 [3]. With improvement in technology and surgical technique, laparoscopic ventral hernia repair (LVHR) has gained significant popularity and is now routinely performed in most centres. Certain benefits offered by laparoscopic surgery include shorter hospital stay and reduced postoperative pain and studies have also reported fewer complications in laparoscopic surgery as compared to open surgery [4].

The primary objective of our study was to compare open ventral hernia repair with laparoscopic repair in relation to postoperative pain while our secondary objectives were to compare the duration of surgery, hospital stay, and return to normal activity between the two groups.

## Materials and methods

**Figure 1:**
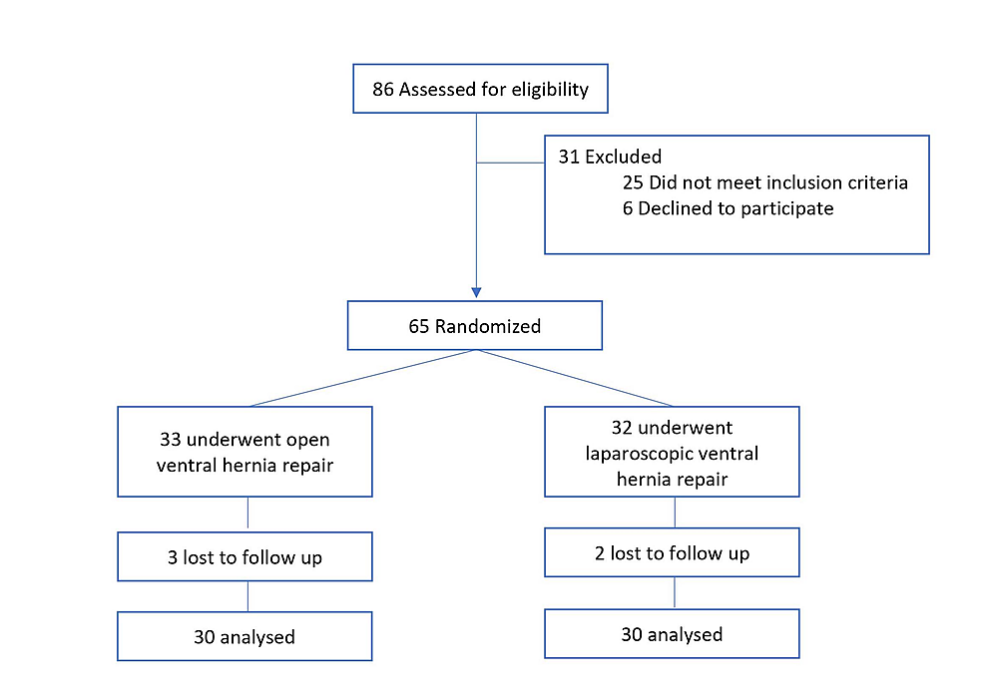
CONSORT Diagram CONSORT: Consolidated Standards of Reporting Trials

This prospective study was conducted from August 2015 to December 2017 at Father Muller Medical College Hospital, Mangalore. Father Muller Institutional Ethics Committee issued approval FMMC/FMIEC/1864/2014. Inclusion criteria included patients with non-complicated ventral hernias. Exclusion criteria included those unfit for general anaesthesia, recurrent hernias, complicated ventral hernias, and those converted from laparoscopic to open surgery. Out of the 86 patients found eligible for the study, 31 were excluded as they did not meet the inclusion criteria or declined to participate. The remaining 65 patients were divided into two groups. Those in group I underwent open ventral hernia repair while patients in group II underwent LVHR. Simple randomization using computer-generated numbers were used to classify patients into two groups. Patients and medical staff were not blinded to the allocated procedure. Three patients in the open group and two patients in the laparoscopic group were lost to follow-up leaving behind 30 patients each in the open and laparoscopic groups for analysis (Figure 1).

Operative technique

Laparoscopic Repair

Pneumoperitoneum was created using a Veress needle. A 10mm port and two or three 5mm working ports were placed based on the site of the hernia. After reduction of hernial contents, a dual mesh was placed with a 5cm overlap beyond the margins of the defect. The mesh was secured to the anterior abdominal wall with metallic tacks. In larger defects, the mesh was first secured using transfascial sutures. The skin was closed by staples.

Open Repair

The skin incision was made based on the site of the hernia. The hernial sac was dissected out and contents were reduced. The primary defect was closed with Prolene 1-0 suture. Subcutaneous flaps were raised to about 5cm beyond the defect. A Prolene mesh of adequate size was placed over the site of defect and was then secured to the anterior rectus sheath with Prolene sutures. The skin was closed with nylon sutures over a suction drain.

Postoperatively, all patients received intramuscular diclofenac for 48 hours followed by oral diclofenac as and when required. Pain experienced by patients was recorded using the visual analogue scale (VAS) on the first and seventh postoperative days. Patients were encouraged to start oral feeds eight hours following the surgery, initially with liquids followed by a normal diet. Surgical wounds were inspected on the day of discharge for seroma and signs of infection. Drains were taken out when output was less than 30ml. Patients were initially followed up weekly for the first month followed by monthly for the next five months. Operative time, postoperative complications, postoperative pain, duration of hospital stay and time to return to normal activity after discharge were recorded. In order to avoid bias, all surgeries were performed by two experienced surgeons. Institutional ethics committee approval was taken before commencing the study.

Data were analysed using IBM SPSS Statistics for Macintosh, Version 21.0 (Released 2012, IBM Corp., Armonk, New York). Qualitative data were presented as numbers and percentages while quantitative data were presented as mean ± standard deviation. Data were analysed using student’s t-test and Chi-square test and a p-value of less than 0.05 was considered to be statistically significant.

## Results

Mean age of patients in the open group was 52.1 ± 11.51 years while the mean age in the laparoscopic group was 43.20 ± 9.37 years. There were equal number of males and females in both the open and laparoscopic group (Table 1).

**Table 1:** Patient Demographics

	Open Group (n = 30)	Laparoscopic Group (n = 30)
Age (mean ± SD)	52.1 ± 11.51	43.20 ± 9.37
Sex		
Male	12	12
Female	18	18

Paraumbilical hernias were the most common in both groups, which accounted for 71.6% of all patients involved in our study. Incisional hernias were the second most common (21.7%) followed by epigastric hernias, which were the least common (6.7%) (Table 2).

**Table 2:** Types of Ventral Hernias

Type of Hernia	Open group (n = 30)	Laparoscopic group (n = 30)	Percent (%)
Epigastric Hernia	2	2	6.7%
Incisional Hernia	6	7	21.7%
Paraumbilical hernia	22	21	71.6%

Preoperative defect size was equal in both groups with a median of 4 cms. The defect in the open group had an interquartile range of 2-8 cms while in the laparoscopic group it ranged from 2-6 cms (p=0.41).

The mean operative time was significantly longer in the laparoscopic group (116 ± 4.1 min) as compared to the open group (67.3 ± 2.3 min). Pain experienced by the laparoscopic group on the first postoperative day based on VAS was less as compared to the open group. On the seventh postoperative day, the majority of the patients in the laparoscopic group experienced grade 1-2 on VAS as compared to the open group that experienced grade 2-3. The mean duration of postoperative stay in the open group was 6.23 days, which was longer as compared to the laparoscopic group (3.17 days). Patients in the laparoscopic group resumed normal activity quicker as compared to the open group (Table 3). 

**Table 3:** Operative Outcome

	Open Group (n=30)	Laparoscopic Group (n=30)	p-value
Operative time (minutes) (mean ± SD)	67.3 ± 2.3	116 ± 4.1	<0.01
Pain score on first postoperative day (mean ± SD)	6.31 ± 1.33	3.14 ± 1.41	<0.01
Pain score on seventh postoperative day (mean ± SD)	2.1 ± 1.22	1.13 ± 2.3	0.01
Duration of hospital stay (days) (mean ± SD)	6.23 ± 0.35	2.17 ± 1.12	0.02
Return to normal activity (days) (mean ± SD)	2.87 ± 0.34	1.47 ± 0.11	<0.01

## Discussion

There are numerous surgical techniques to repair ventral hernias. In the past, simple suture repair was performed, which was associated with a high rate of recurrence [5]. The earliest report of the use of a prosthesis for ventral hernia repair was in 1958 [6]. Since then, recurrence rates associated with ventral hernia repair has significantly reduced, but there still exist debates as to which type of procedure is most appropriate for the treatment of ventral hernias.

We had a greater number of female patients in our study in both the open and laparoscopic groups as compared to males. Basheer et al. [7] also had a greater number of females in their study, which was believed to be due to higher cosmetic concerns in the female group. LVHR had a significantly longer operative time as compared to open repair in our study. This may be due to the time taken to set up the laparoscopic equipment as well as achieving adequate pneumoperitoneum prior to starting the definitive repair. Basheer et al. [7] in their study found the operative time in LVHR shorter as compared to open repair. Open repair was longer in their study mainly due to the time required to raise flaps prior to mesh placement in open repair. Other studies also found that open repair took a longer time as compared to LVHR [8,9].

Pain experienced by patients was assessed based on the VAS. On the first postoperative day, the majority of patients in the laparoscopic group experienced less postoperative pain as compared to the open group. We also found that on the seventh postoperative day patients with open repair scored a two on VAS as compared to the laparoscopic group that scored a one. Navarra et al. [10] had similar findings to our study. They also found that the duration of postoperative analgesia was significantly longer in the open group (4.9 days) as compared to the laparoscopic group (1.4 days).

In terms of length of hospital stay, the open group was longer in comparison to the laparoscopic group. The main factor that contributed to the longer hospital stay was the pain experienced by patients in the open group. Basheer et al. [7] had similar findings to our study where the mean duration of hospital stay in the laparoscopic group was 1.15 days as compared to 4.55 days in the open group. These findings were quite consistent with other studies by Olmi et al. [11], Navarra et al. [10] and Mishra et al. [12].

In our study, patients resumed normal activity by the first day following discharge from the hospital as compared to the open group, which took longer. Basheer et al. [7] found that patients with open repair took a considerably longer time to return to normal activity (13.8 days). We inspected the wounds for infection prior to discharge from the hospital and none of the patients had any signs of surgical site infection. At the two-week follow-up, two patients in the open group had developed a small seroma that was managed by aspiration. No such complications were recorded in the laparoscopic group. One of the limitations of our study is that there was no blinding when assessing the postoperative pain in the two groups. The other limitation was that we did not take patient comorbidities into consideration as this could potentially increase the risk of postoperative complications.

## Conclusions

LVHR is routinely performed in most centres and is a safe and feasible alternative to open ventral hernia repair. Our study showed that LVHR has a significant advantage over open repair with respect to postoperative pain, hospital stay, and return to normal activity.
